# Thought-Controlled Nanoscale Robots in a Living Host

**DOI:** 10.1371/journal.pone.0161227

**Published:** 2016-08-15

**Authors:** Shachar Arnon, Nir Dahan, Amir Koren, Oz Radiano, Matan Ronen, Tal Yannay, Jonathan Giron, Lee Ben-Ami, Yaniv Amir, Yacov Hel-Or, Doron Friedman, Ido Bachelet

**Affiliations:** 1 Efi Arazi School of Computer Science, The Interdisciplinary Center, Herzliya, Israel; 2 Advanced Virtuality Lab, Sammy Ofer School of Communications, The Interdisciplinary Center, Herzliya, Israel; 3 Faculty of Life Sciences and the Nano-Center, Bar Ilan University, Ramat Gan, Israel; Duke University, UNITED STATES

## Abstract

We report a new type of brain-machine interface enabling a human operator to control nanometer-size robots inside a living animal by brain activity. Recorded EEG patterns are recognized online by an algorithm, which in turn controls the state of an electromagnetic field. The field induces the local heating of billions of mechanically-actuating DNA origami robots tethered to metal nanoparticles, leading to their reversible activation and subsequent exposure of a bioactive payload. As a proof of principle we demonstrate activation of DNA robots to cause a cellular effect inside the insect *Blaberus discoidalis*, by a cognitively straining task. This technology enables the online switching of a bioactive molecule on and off in response to a subject’s cognitive state, with potential implications to therapeutic control in disorders such as schizophrenia, depression, and attention deficits, which are among the most challenging conditions to diagnose and treat.

## Introduction

Controlled drug delivery systems aim at improving the spatial and temporal resolution of therapeutic molecules. For example, linking a drug to an antibody could render it highly selective to the antibody’s cognate epitope, a concept already used in practice. However, the temporal dimension is significantly more challenging. Drugs can be embedded in a matrix, such as a particle or liposome, from which they diffuse slowly into the bloodstream or tissue[1], and various technologies combine this concept with spatial targeting [2]. However, until recently, no system could provide explicit temporal control of a drug, e.g. generating a sequence of alternating activation/inactivation of the drug for arbitrary periods of time. Any system that releases drugs, regardless of release kinetics, is inherently irreversible and therefore does not provide true therapeutic control (in the engineering sense of the term ‘control’).

Recently, drug-loaded nanoscale robots were reported, which would not release drug molecules, but rather reversibly expose and conceal them to the environment while maintaining them physically linked to their chassis[3]. These nanorobots are the first system that approaches real arbitrary control of therapeutic molecules; however, it requires comprehensive knowledge of molecular targets discriminating healthy from abnormal environment states. While in many diseases, such as cancer, these targets are well defined, other diseases are much more difficult to characterize in molecular terms of good and bad, making them extremely elusive to diagnose and treat. Mental disorders such as schizophrenia, depression, attention deficits, and autism, exemplify this challenge. These conditions therefore require different modes of control over therapeutic molecules, which are driven by patient’s mental and cognitive states.

In the last two decades there has been extensive progress in brain-computer interfaces (BCIs), allowing both healthy and disabled individuals to control a wide range of devices using mental activity alone[4,5]. The mental activity is decoded from brain activity by applying a combination of signal processing and machine learning techniques to various neurophysiological signals, recorded invasively or non-invasively. Such BCIs allow people to move a cursor on a screen[6], navigate in virtual reality[7], control robots[8], robotic prostheses[9,10], and more. Real-time brain mapping technologies are also suggested in assisting the diagnosis and treatment of mental disorders, providing a new communication and control technology for disabled individuals and the general population[11,12,13]. Nevertheless, so far no interface has been established between a human mind and a therapeutic molecule, which are 10 orders of magnitude apart. The purpose of this study was to show that DNA robots can bridge this gap.

To establish a direct control interface to DNA robots, we designed robots that can be electronically remote-controlled. This was done by adding metal nanoparticles to the robotic gates, which could heat in response to an electromagnetic field. This concept has been demonstrated previously[13], and has been recently implemented in controlling gene expression in an animal model of diabetes[14].

In this paper we integrate all these components to allow EEG patterns associated with cognitive states to remotely trigger nanorobot activation in a living animal, and describe the design, construction, and implementation of this brain-nanomachine interface. Our working prototype highlights the potential of such a technology in managing disorders to which no effective treatment exists, and could inspire advanced modes of control over biological molecules in the body even outside therapeutic contexts.

## Methods

### Experimental setup

The full experimental setup consisted of five components: a) a headset used for collecting EEG data from the subject; b) an algorithm that searches for patterns associated with cognitive load and rest states, running on a computer; c) a waveform generator, remote controlled by the computer, which produces high-frequency alternate current through the coil; d) the coil itself; and e) the DNA origami robots, injected into the living animal fitted within the coil. Data collection was carried out separately from this setup, and included only the headset connected to a computer (see **Data collection** below).

### Data collection

Recording of EEG signals was conducted using 4 g.LADYbird sintered Ag/Cl crown active ring electrodes located on the subject’s frontal lobe at PZ, FZ, AF3 and AF4 locations according to the international 10–20 system. Reference electrode was positioned on the subject’s right ear lobe and ground electrode was placed at Fpz location according to the international 10–20 system. EEG signals were recorded at 256 Hz sampling rate, with amplification, analog filtering (5–100 Hz) and notch filtering (50Hz) performed using g.USBamp amplifier (Guger Technologies, Schiedlberg, Austria). Data was stored on an HP PROBOOK laptop as generic matrix (.mat) files for further analysis. EEG electrode placement was done using a g.GAMMAcap EEG cap (Guger Technologies, Schiedlberg, Austria), each electrode designated space between the electrode and the participants scalp was filled with g.GAMMAgel (Guger Technologies, Schiedlberg, Austria) in order to assure high signal conduction. Reference electrode was attached to the right ear lobe using the built in clip provided with the electrode. Six features (RMS, Theta, Alpha, Low Beta, High Beta, and Gamma) were extracted from the raw EEG data using Fast Fourier Transform (FFT).

### SLACC algorithm design

For algorithm training, feature data from the raw EEG readings of 7 subjects was manually classified into two classes (cognitive rest and cognitive load) and then reduced from FFT-derived 24D vectors to 2D by linear discriminant analysis (LDA). LDA output, represented by a 2D projected vector, was distributed into 4-second windows, with 3 second overlap between them. Each window was subsequently entered into a support vector machine (SVM) for training, resulting in SVM distinction between the two classes. Data from the actual experiment was processed in the same way through LDA before being entered into SVM for query and decision. A more detailed description of algorithm design and performance can be found in S1 File.

### DNA origami robots

Robots were designed with caDNAno 2.0[15] (caDNAno files are available in the supporting information to this paper), using the M13mp18 bacteriophage ssDNA as a scaffold strand. Staple strands were synthesized by Integrated DNA Technologies (IDT). Folding was carried out in folding buffer (1X Tris-Acetate-EDTA supplemented with 10 mM Mg^2+^) on a standard thermal cycler according the following annealing sequence: 80°C to 61°C at 5 min/°C, and then 60°C to 25°C at 60 min/°C. Folded robots were partially purified by a single round centrifugal gel filtration on an Amicon Ultra column with molecular weight cutoff of 100 KDa. NHS-functionalized iron oxide nanoparticles were covalently attached to the robots using the internal amino-deoxythymidine residues introduced in the design process, by mixing nanoparticles and robots at a nanoparticle molar excess of 100:1, followed by two more rounds of purification. Prior to injection, robots were exchanged into phosphate buffered saline containing 10 mM Mg^2+^. Antibody Fab’ fragment payload was prepared by cleaving whole IgG molecules using a commercially available Fab’ preparation kit according to the manufacturer’s instructions. The antibody fragments were tagged by amine-reactive DyLight 650, purified and chemically conjugated to amine-modified U1c DNA (5’-NH_2_-GAACTGGAGTAGCACAA-3’) using EDC conjugation. Following purification of the product, the payload fragments were mixed with robots at a molar excess of 20:1 (payloads over robots) for at least 1 hour at room temperature, prior to addition of guide removal strands and a final purification round of loaded robots.

### Animals

Adult *B*. *discoidalis* insects were purchased from Meital Labs (Israel) and maintained in red soil-layered plastic containers. Empty egg cartons were used as light shelters, with food (dry dog food and fresh fruit) and water (moist cotton wool in a plastic vessel) kept fresh and provided ad-libitum. The maximum number of insects per container was 12. After an initial adjustment period during which approximately 1 of every 8 insects died, the insects were maintained for up to 6 months without evident signs of stress or further deaths. The containers were monitored at all time using a web video camera connected to a DVR that stored the last 14 days of visual data.

### Ex vivo/in-vivo experiments

Injections were performed in a sequence and not in parallel, to minimize animal stress. Each insect in turn was incubated in a clean glass beaker at -20°C for 7–10 min, depending on its size and behavior. Robots (100 fmol in 10 μL phosphate buffered saline containing 10 mM Mg^2+^) were injected using a Hamilton syringe to the insect’s abdomen in a flat angle through the membrane between the two apical sternites. After the injection, the insects were inserted into the coil such that the abdomen was exactly in the middle of the coil’s length. The custom-built coil was connected to a Rohde-Schwartz SML02 signal generator, producing a 14.6 MHz signal at 50 W such that an electromagnetic field of 5 mT was generated inside the coil. The Hamilton syringe was cleaned with water and 70% ethanol in between injections. After finishing the experimental sequence, the animal was lightly anesthetized again, and hemolymph was extracted by puncturing the left arthrodial membrane using a G18 needle pre-wetted with anticoagulant buffer (30 mM citric acid, 30 mM sodium citrate, 1 mM EDTA, 0.05% w/v NaN_3_), and aspirating the exudate directly into an ice-cooled pipette tip filled with 25 μL cold anticoagulant buffer. The exudate was then diluted 1:1 into ice-cold anticoagulant buffer and analyzed by flow cytometry. For ex-vivo experiments, hemolymph cells were isolated from untreated, lightly anesthetized animals by puncturing and aspirating exactly as described above.

### Flow cytometry

Flow cytometry was performed on an Accuri C6 flow cytometer equipped with a 488 nm solid-state laser and a 640 nm diode laser. Data were coarse-analyzed using FlowPlus software followed by analysis on FCS-Express 4.0 software (using a C6 import module).

## Results and Discussion

The nanoscale DNA robots used in this study were a modified version of the nanorobot we described previously. Providing the nanorobots with the ability to respond to electromagnetic fields enables autonomous sensing of biomolecules and possibly computational abilities [3, 14]. A crucial advantage of these robots is that they integrate a solid shell, fabricated from DNA origami, with gates made of complementary DNA strands. The shell can be reversibly closed or opened by controlling gate strand hybridization, using strand displacement, aptamer binding, etc. When the shell is closed, it sequesters molecular or nanoparticulate payloads of various types up to a size of 25 nm, such that they are concealed from the environment. Opening of the shell exposes these payloads, which remain stably tethered to the shell and are not released. Thus, by controlling the shell, it is possible to reversibly switch therapeutic molecules between exposed/concealed states equivalent to on/off states, respectively. As the robots do not enter cells efficiently and remain mostly extracellular, they can only support payloads that engage cell surface targets. This seems like an inherent drawback of the system; however it should be noted that any therapeutic outcome could arguably be achieved from outside the cell, since almost all endogenous signals operate through cell surface receptors (with exceptions such as steroid hormones and nitric oxide).

The second goal was to control DNA remotely from outside the host’s system. Therefore, we re-designed the robots to enable the addition of functionalized metal nanoparticles, which could be heated by applying a radio frequency-induced electromagnetic field (RFMF) on the entire animal. Few previous studies demonstrated remote control over DNA hybridization using gold nanocrystals[16], however only in-vitro. In contrast, many works reported the in-vitro and in-vivo heating of metal nanoparticles for purposes of tumor killing, control of enzymatic activity, drug delivery, or inducing gene expression[17],[18],[19]. As an animal model for this proof of concept we chose the insect *B*. *discoidalis*, which was recently employed as a model for molecular control by DNA origami, showing high compatibility and reproducibility[14].

**Fig 1:**
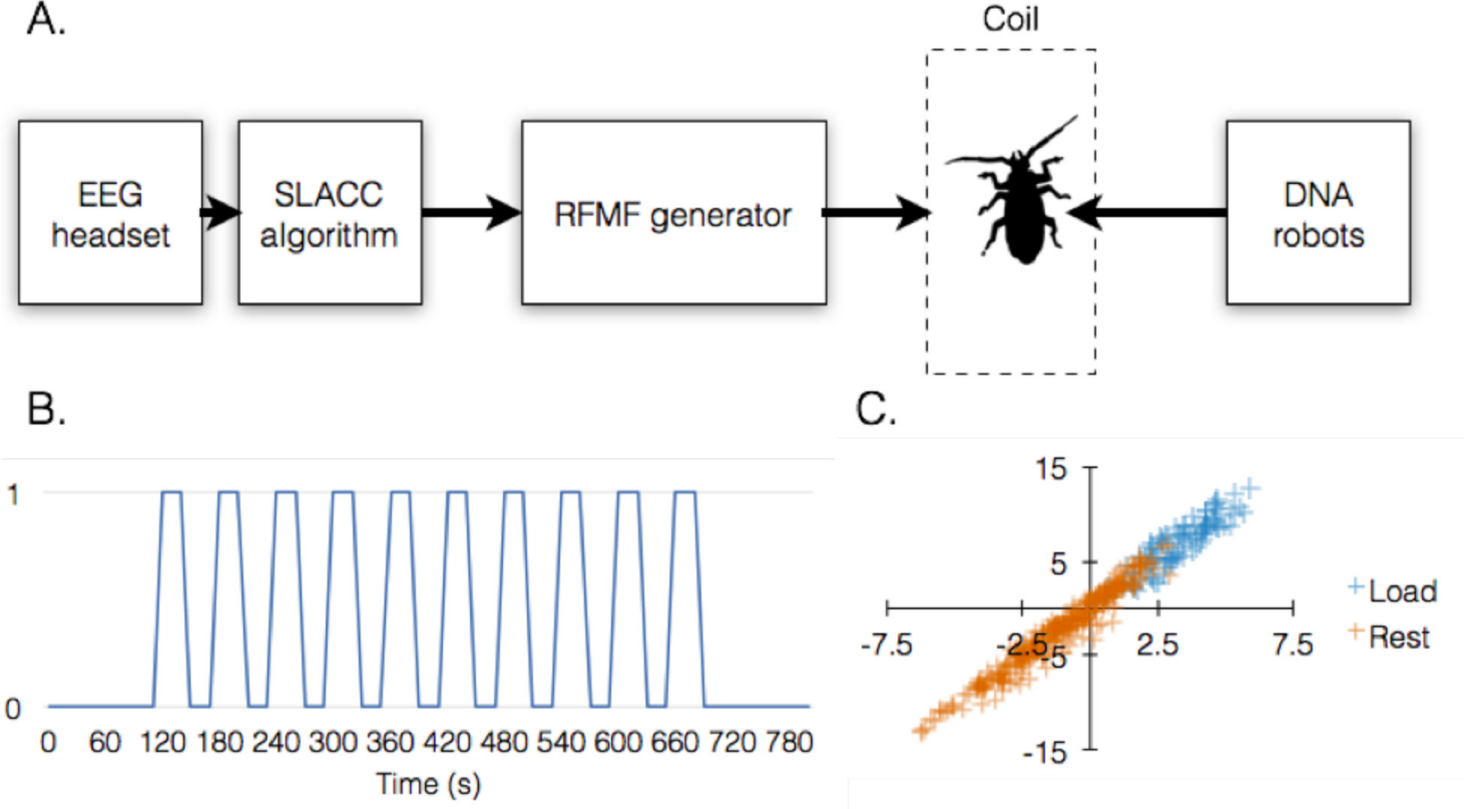
System outline and performance. (A) Basic system outline: signals recorded by the EEG headset on the subject were monitored by the SLACC algorithm, which controls the state of an RFMF generator connected to an induction coil. The test animal is placed inside the coil after being injected with DNA robots. (B) Experimental protocol structure. 0 and 1 on the Y-axis denote states of cognitive rest and load, respectively, which are induced by displaying alternating screens showing either nothing or a list of arithmetic problems **(S1 File)**, respectively. (C) Classification of cognitive rest vs. cognitive load signals by SLACC.

As a proof of principle we focused on driving the activity of DNA robots inside a living animal by an induced state of cognitive load in the test subject. To induce cognitive load, we compiled a test from simple arithmetic problems, which the subject was required to solve during the defined cognitive load phase of the experiment. The test was projected onto a screen in front of the subject, followed by a screen designed to move the subject back to cognitive rest. We then designed and trained a pattern classification algorithm, termed SLACC, to discriminate on-line between these two states in the raw EEG data recorded from the subject. SLACC achieved a precision of 92.5% and sensitivity of 86.3% (Fig 1A–1C). A detailed description and performance analysis of this algorithm can be found in S1 and S2 Files.

**Fig 2:**
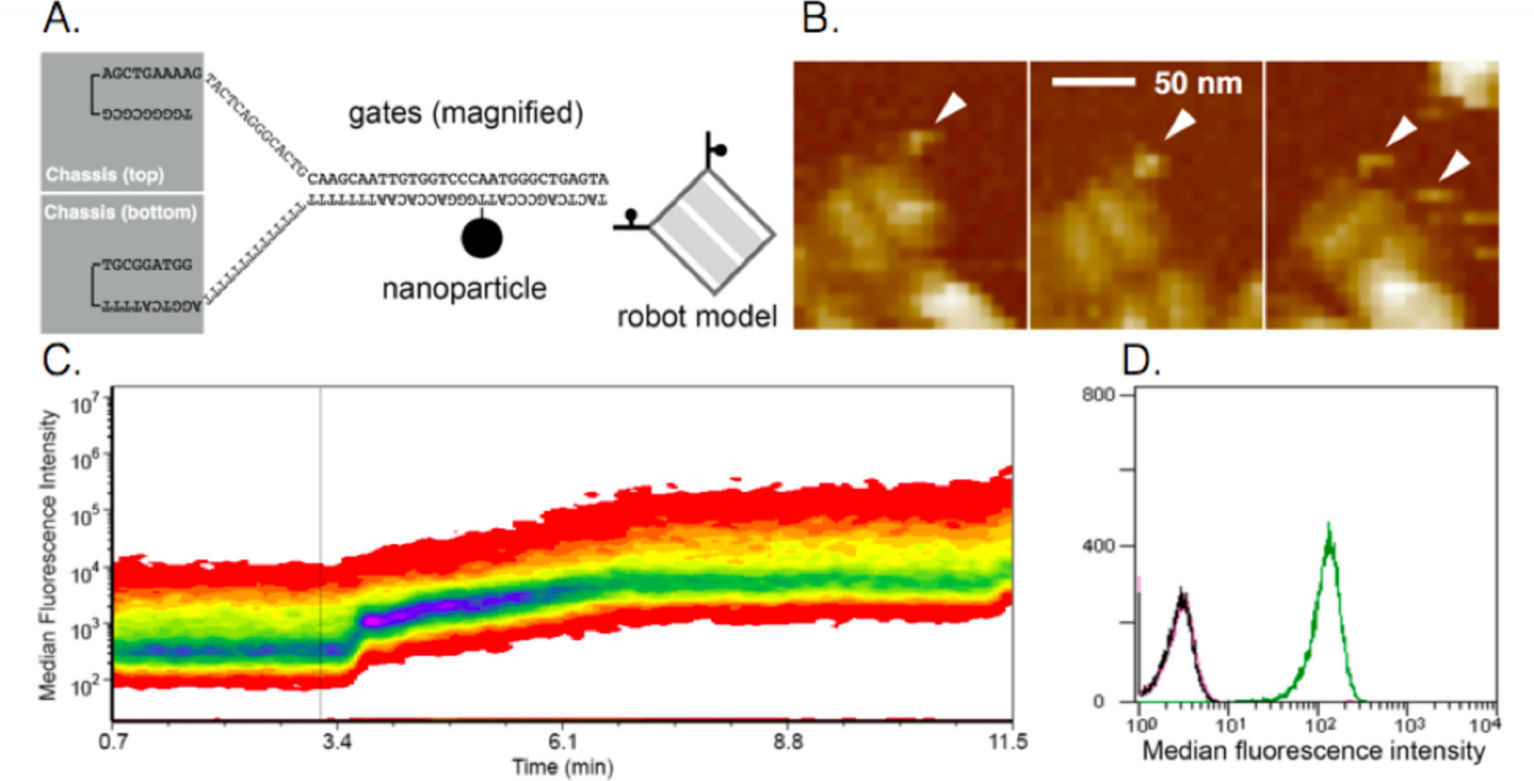
Remote-controlled DNA robot design and performance. (A) schematic showing the link between robot and metal nanoparticle, on a magnified portion of the gate strand. A nanoparticle is chemically linked to one strand of each gate as shown in the robot model. (B) AFM images of robots conjugated with nanoparticles. (C) RFMF-induced activation of robots and subsequent staining of target cells with fluorescent antibodies loaded inside the robots. Fluorescence is monitored in real-time by flow cytometry. (D) Flow cytometric validation that transient RFMF-induced activation does not destroy the robots (black, closed robots; green, open robots; magenta, transiently RFMF-activated robots).

We chemically attached iron oxide nanoparticles to each gate of the robots (Fig 2A and 2B), and loaded the robots with fluorescent antibody fragments recognizing surface epitopes on the cells of the insect *B*. *discoidalis*. The suspended cells were then placed inside an induction coil through which a 14.6 MHz RFMF was applied by a signal generator. The signal generator was activated only when the test subject’s EEG pattern was recognized by our classification algorithm as cognitive load. RFMF activation induced robot opening and subsequent cellular staining by the exposed fluorescent antibody fragments which was tracked in real-time. An increase in cell fluorescence was observed as early at t = 18s following field activation (t = 0), which peaked rapidly until t = 42 s and continued to increase slowly, reaching maximal level at t = 3.8 min (Fig 2C). The robot gates have a predicted melting temperature (*T*_m_) of 56°C. However, as previously described, these gates hold together the two parts of the robot shell, each with a mass of 2.4×10^6^ Da, imposing an entropic penalty to the hybridized gate strands, rendering them less stable and more leaky than predicted by *T*_m_ alone. Therefore, the increase in temperature (ΔT) required for robot opening is likely smaller than that required for reaching the predicted *T*_m_. Assuming a ΔT of 15°C, we estimate that the gate strands were heated by the metal nanoparticles at an approximate rate of 0.25°C/s. Importantly, robots that were heated by a 5-min RFMF and allowed to re-close following field deactivation did not stain the insect cells (Fig 2D), confirming that heating for 5 min did not melt the robot shell. This observation is in agreement with a recent report showing that the melting of 3D DNA origami structures occurs at a critical temperature that is ~10°C higher (depending on specific shape) than the critical temperature at which folding occurs[20]. This allows a protection margin for heating and reversible payload exposure before the robots likely melt.

**Fig 3:**
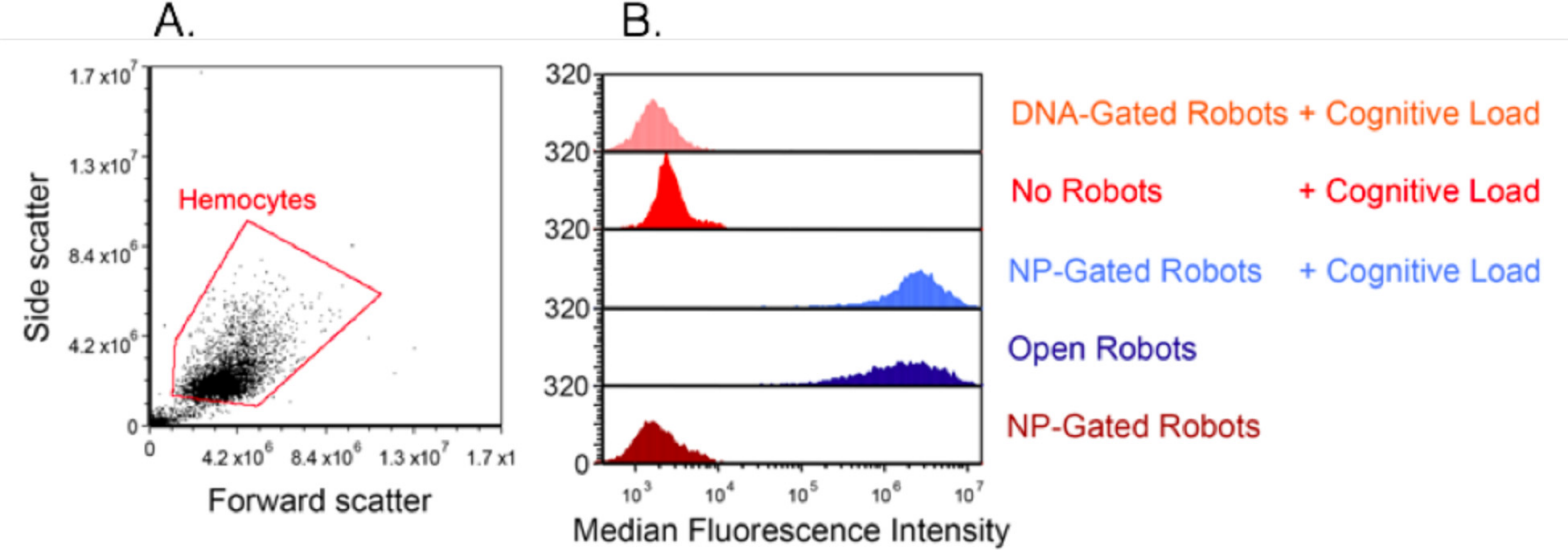
EEG-activated robots engage insect hemocytes in-vivo. (A) physical parameter analysis of *B*. *discoidalis* hemocytes following their extraction after each separate experiment. (B) results from five experimental groups: no robots (red), nanoparticle (NP)-gated robots without cognitive load (brown), NP-gated robots with cognitive load (light blue), DNA-gated robots (no NP) with cognitive load (orange), and open robots as a positive control (dark blue).

The system was integrated by connecting the signal generator to the human subject through the computer on which the algorithm operates. The robots were injected to adult *B*. *discoidalis* and the animals were inserted into the coil. Following a 1 min recuperation period, the test began. During the test, the subject’s EEG was acquired and monitored by the algorithm, which controlled the RFMF generator in real-time. The experiment was repeated separately five times. At the end of each experiment, insect hemocytes were extracted and analyzed for fluorescence (Fig 3A). This analysis showed that robots bearing metal nanoparticles opened and engaged the cells efficiently, generating signals comparable to those generated by constitutively-open robots. In contrast, robots not bearing nanoparticles, or those that bear nanoparticles but were not loaded with fluorescent antibody, did not generate any visible signal (Fig 3B). These findings demonstrate a successful interface between the test subject and the DNA robots inside the living animals.

We chose brain activity as the signal of choice in our paradigm but that does not necessary mean it is the most suitable signal; it is but one chosen physiological parameter with which we decided to demonstrate this system. Moreover, just as BCI technology is becoming more widespread and accessible, so are heart monitoring applications for mobile devices, and in the future even parameters such as blood glucose.

The present study is merely a demonstration and proof of concept for integrating physiological output with molecular control. And here is also the value of this work–in our hope that it will highlight the possibility for such new therapeutic strategies, and encourage building on such drafts in order to achieve optimal designs.

Albeit a very preliminary prototype, this system could inspire improved designs towards thought-mediated control over biochemical and physiological functions assisted by biocompatible molecular machines.

## Supporting Information

S1 File SLACC algorithm design. (PDF)

S2 File SLACC performance analysis. (PDF)
