# Supplementary material for: Thought-Controlled Nanoscale Robots in a Living Host
Source: PLoS One. 2016 Aug 15;11(8):e0161227. doi: 10.1371/journal.pone.0161227 (PMC4985062; doi:10.1371/journal.pone.0161227)
Supplement: S1 File — (PDF) [file pone.0161227.s001.pdf]

S1 note: Algorithm Design

The SVM-LDA Algorithm for Cognitive Classification (SLACC) is a supervised learning algorithm, which receives raw data from EEG readings as an input, and outputs a classification vector $C \in \{0,1\}^t$, where t is the number of seconds in the tested input, $c_i = 1$ if the ith second was classified as "cognitive load", and $c_i = 0$ if it was classified as "cognitive rest". Furthermore, a "score" can be calculated for a time interval I, to avoid an irregular classification over large periods of time, denoted Si. SLACC is composed of two major segments: (1) a training process that trains the SVM to distinguish "cognitive load" from "cognitive rest"; and (2) a testing process, which classifies unlabeled data according to the SVM model. The algorithm outline is shown as a pipeline in Fig 1, followed by an elaborate description of each segment.

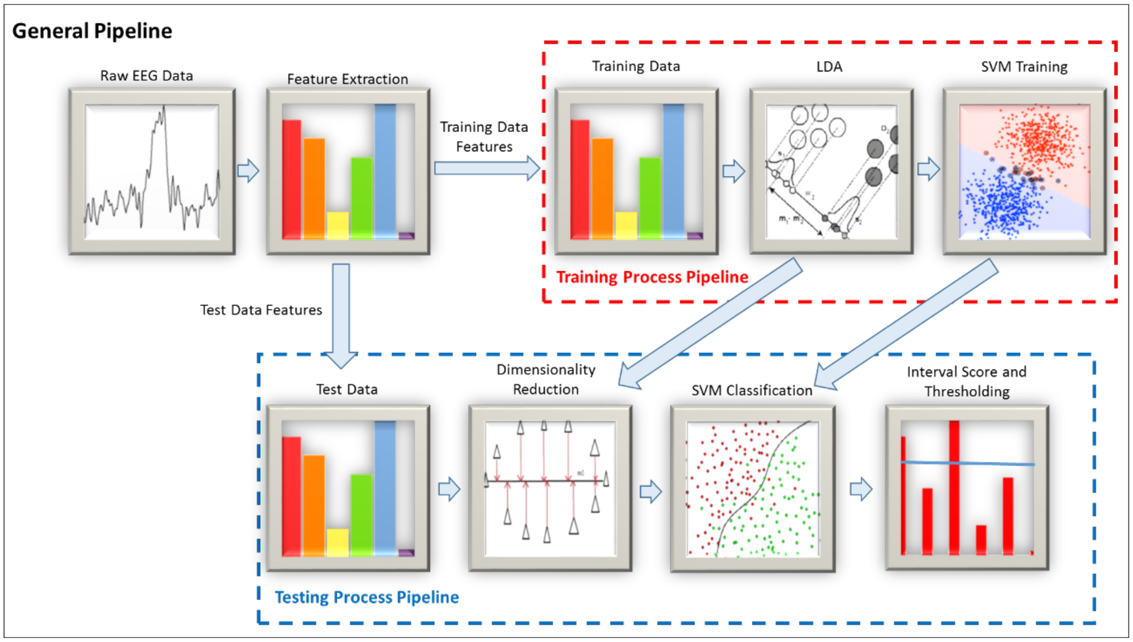

Fig 1: An outline of the SLACC pipeline.

The raw EEG data collected, as described in Section X.Y**,** is processed into six features for each of the four electrodes. Five features were extracted using the fast Fourier transform (FFT) – theta (4-7Hz), alpha(8-12Hz), low Beta(13-21Hz), high Beta(22-30Hz), and gamma(30-100Hz) – along with the root mean square (RMS). These features are extracted over a 4 second running window, calculated every second (resulting in a 3-second overlap between windows, as illustrated in Fig 2). The result is a 24×1024 matrix of featured data for every running window,

concatenated with all other running windows in chronological order. We denote this $24{\times}1024t$ matrix X.

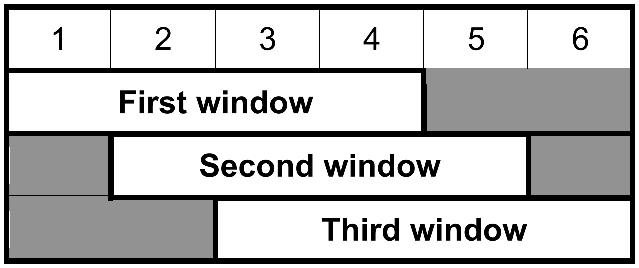

Fig 2: running window illustration

Training stage: Data from X is allocated for training, denoted X_{train}, along with a corresponding vector y, which is a label for each window of the training data (computed manually in accordance with the data collection protocol), i.e., every window of X_{train} is labeled "1" or "0" in the corresponding element of y. Dimensionality reduction for X_{train} from 24 dimensions to 2dimensions is implemented using LDA. We use two Eigen vectors computed by the LDA, denoted v_1, v_2. Dimensionality reduction is achieved by projecting X_{train} on v_1, v_2. We denote the result $X_{train}^{(2)}$. The training data $X_{train}^{(2)}$ and the labels y are transferred to the SVM for a training process, which results in a model discriminating "cognitive load" (1) and "cognitive rest" (0).

Testing stage: The test data, X_{test}, is projected on $v_1$and v_2 from the training stage, and the result, denoted $X_{test}^{(2)}$, is sent to the trained SVM for classification. The classifier output is a vector C, which indicates the SVM classification of each second of $X_{test}^{(2)}$. In order to avoid erratic classification caused by low precision or high sensitivity, the vector C can be augmented into time intervals larger than 1 second, denoted $I_1, I_2, \dots, I_t$. Once performed, a score $s(C, I_j)$ can be calculated for each interval, where: $s\left(C, I_j\right) = \frac{\sum_{i \in I_j}(c_i)}{|I_j|}$, i.e. the sum of all 1's in interval I_j, divided by its length in seconds. We note that $s\left(C, I_j\right) \in [0,1]$, and by assigning a threshold parameter h we can classify "cognitive load" for an interval I_j if $s\left(C, I_j\right) \geq h$, or "cognitive rest" for $s\left(C, I_j\right) < h$, and circumvent inconsistent 1 second classification.
