# Supplementary material for: Thought-Controlled Nanoscale Robots in a Living Host
Source: PLoS One. 2016 Aug 15;11(8):e0161227. doi: 10.1371/journal.pone.0161227 (PMC4985062; doi:10.1371/journal.pone.0161227)
Supplement: S2 File — (PDF) [file pone.0161227.s002.pdf]

S2 note: SLACC performance analysis

SLACC is composed of a Training Process and a Testing Process, which results in a classification of EEG data according to the SVM training. In order to inspect algorithm performance, a k-Fold Cross-Validation method was implemented, where k = 10. The discussion focuses on precision and sensitivity magnitudes for the classification-vector C and the classification-score $s(C,\mathbf{I}_j)$. The ground truth is derived from the experiment outline and protocol, which is designed to make a distinction between "Cognitive-Load" (CL) and "Cognitive-Rest" (CR).

CL was prompted in the seven test subjects by a task composed of simple arithmetic problems, as shown in figure S1. Subjects were instructed to solve these problems without the use of any accessories, in defined limited time frames. Those time frames will be referred to as "load", and correspond to CL in the ground truth of the classification analysis. To induce CR, subjects were shown a blank screen, and were directed to stare and relax. This is indicated as "rest" and represented by CR in the ground truth.

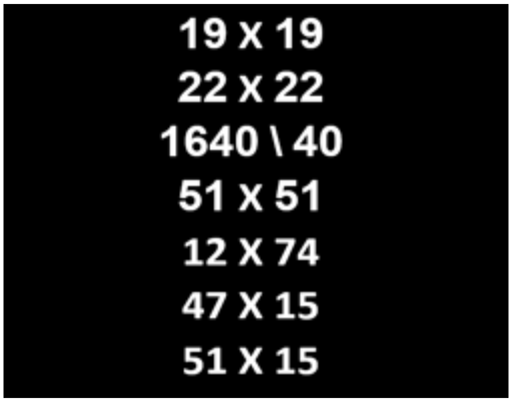

Fig S1. Arithmetic problems for induction of CL

Experiment Protocol

A 13.5 minute protocol was designed and performed by the test subjects, while their EEG emission is documented and analyzed. The first 120 seconds were rest, to enable the subject to relax and focus. That was followed by alternating 30 second frames of load and rest, which summed to 570 seconds (10 load windows, 9 rest windows). The experiment protocol concluded with additional 120 seconds of rest for total experiment duration of 810 seconds. For further clarification, the protocol's ground truth is illustrated in figure S2.

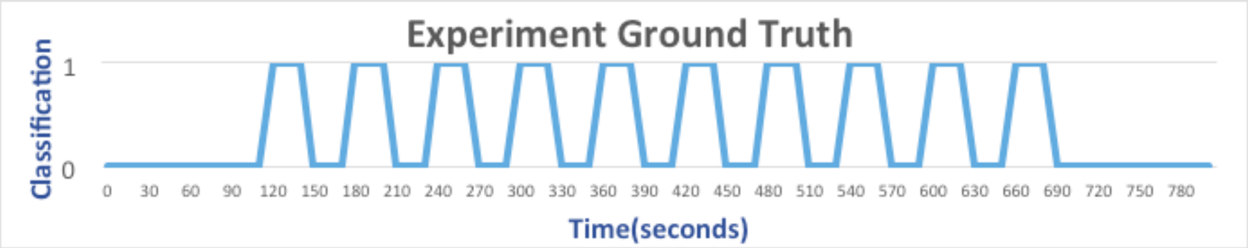

Fig S2. Experimental protocol ground truth.

Performance

The experiment was conducted on seven different subjects. In order to increase the amount of analyzed data, we used k-Fold Cross-Validation technique, which allowed us to examine SLACC's performance several times on each subject. We used k = 10, which in each of the k folds 60% of the data was applied for the training process, and 40% was used as cross-validation. Performance is computed for the interval score classification $s(C,I_j)$ using a threshold h.

After feature extraction is performed on the raw EEG data, the training process executes LDA on the labeled training data, in order to handle simpler 2D information. The result is exemplified in figure S3. The SVM receives the dimensionally reduced data and learns to discriminate CL from CR. Following the training process, the cross-validation featured data is projected upon the 2D plain found by the LDA, and the trained SVM outputs a vector C that indicates for each second of the cross-validation data, whether it was classified as CL or CR, i.e. "1" or "0" respectively. Figure S4 illustrates the training placement and cross-validation classification output of SLACC for every fold of a specific subject. It is noted that there is no apparent difference in the algorithm's performance in each fold, and some false positive and false negative classifications occur, to be discussed later.

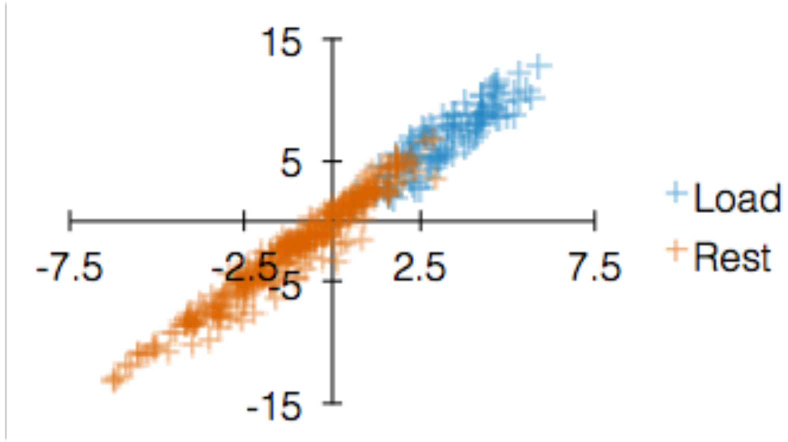

Fig S3

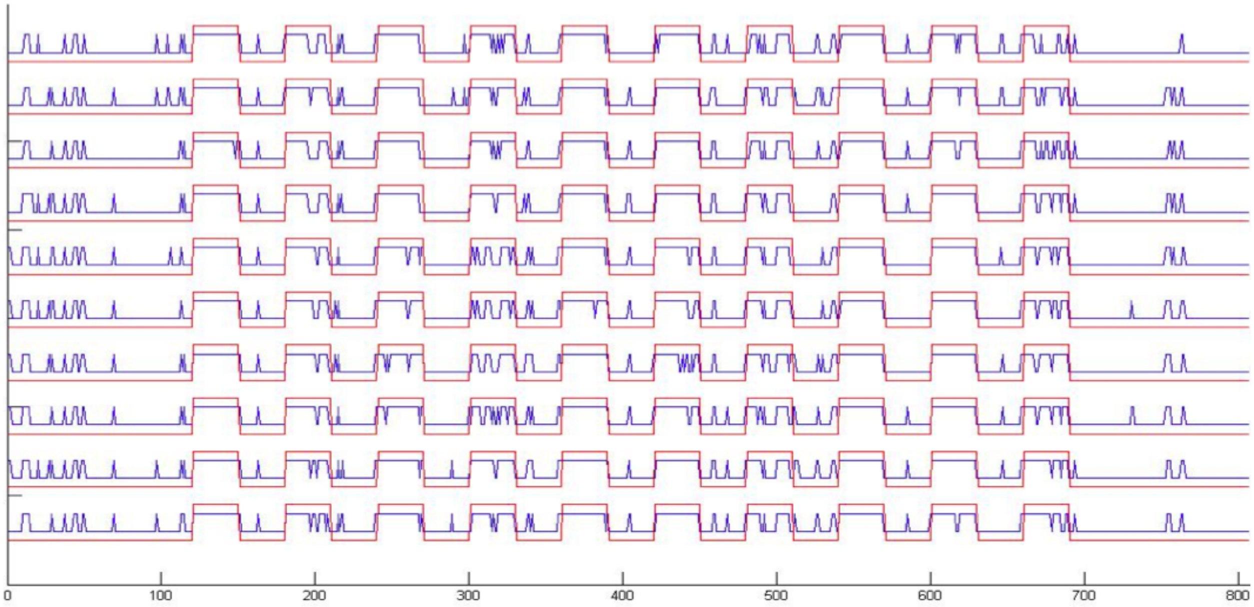

Fig S4. SLACC output classification vector C for 10-fold cross-validation of a specific subject. Red represents ground truth. X-axis denotes time in s.

The interval classification is performed by setting a threshold h, and classify CL if $s(C,I_j)>=h$, or CR if $s(C,I_j)<h$. The threshold h can be computed manually or by simple calculation in accordance to precision and sensitivity demands. Figure S5 exhibits the change in the mean score of 20 second intervals (excluding 5 second shoulders in each window), of all the cross validation data collected in the experiment. It is clearly visible that the load time frames have a much higher mean score value, which indicates that a threshold can be set to comfortably distinguish CL and CR.

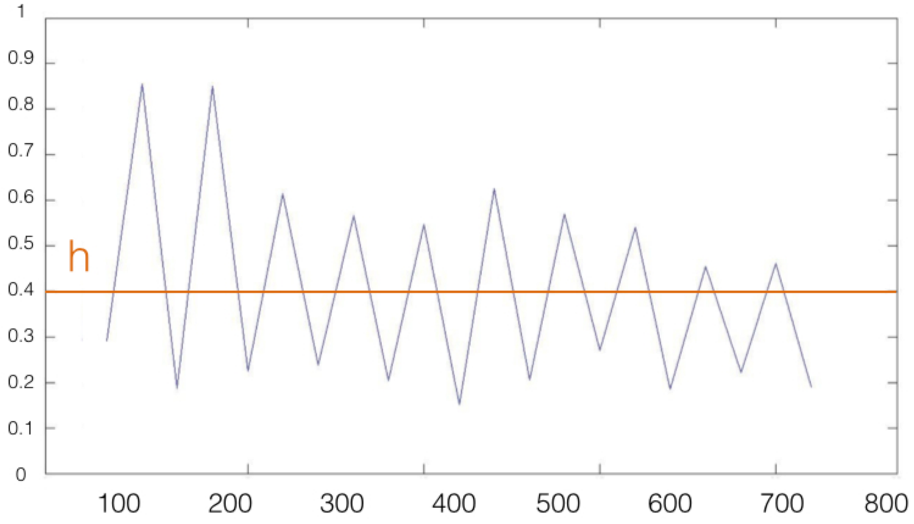

Figure 5: Mean for all subjects in 20 second intervals, removing 5 seconds, with 0.4 threshold h value

Fig S5. Mean for all subjects in 20 second intervals, removing 5 s, with 0.4 threshold h value.

Precision, Sensitivity and F1-Score

In general, due to the nature of the human mind and thought, the vector h provides an erratic and inconsistent classification, which leaves a large room for improvement in SLACC's trustworthiness in this task. As stated before, in order to avoid such irregular classification, a time interval score classification is implemented, along with a threshold, where CL is classified when $s(C,I_j)>=h$. In figure S6, we display performance indicators (precision, sensitivity, specificity, accuracy and F1-score [2*sensitivity*precision/{sensitivity + precision}]) for interval score classification. Clearly, all measurements show consistent and reliable performance, and thus this classification method is taken under account when discussing algorithm performance. Figure S7 shows the ROC curve for the interval score classification, with respect to every test subject. We note that some subjects resulted in very high algorithm accuracy, while others conveyed diminished performance.

F1-score	accuracy	specificity	sensitivity	precision	Subject
1	1	1	1	1	1
0.986	0.988	0.978	1	0.973	2
0.947	0.951	0.911	1	0.9	3
0.909	0.914	0.867	0.972	0.854	4
0.711	0.679	0.511	0.889	0.593	5
0.935	0.938	0.889	1	0.878	6
0.786	0.778	0.667	0.917	0.688	7

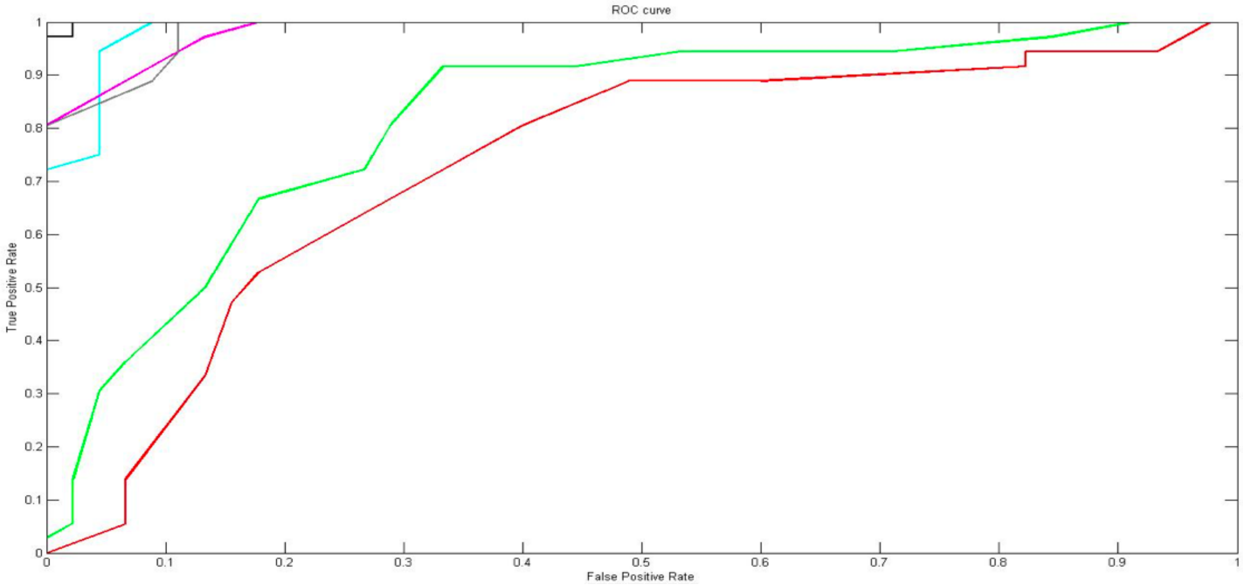

Fig S7. ROC curves for interval score classification.
